# Seroprevalence and risk factors of *Brucella ovis* in domestic sheep in Wyoming, USA

**DOI:** 10.1186/s12917-019-1995-5

**Published:** 2019-07-15

**Authors:** Molly Elderbrook, Brant Schumaker, Todd Cornish, Dannele Peck, Kerry Sondgeroth

**Affiliations:** 1 Department of Veterinary Sciences, University of Wyoming, 1174 Snowy Range Road, Laramie, WY 82070 USA; 2 Northern Plains Climate Hub, United States Department of Agriculture, Agricultural Research Service, 1701 Center Avenue , Fort Collins , CO 80526 USA

**Keywords:** *Brucella ovis*, Sheep, Ram epididymitis, Seroprevalence, Wyoming

## Abstract

**Background:**

*Brucella ovis* causes a sexually transmitted, infectious disease of domestic sheep characterized by genital lesions and epididymitis in rams, placentitis and rare abortions in ewes, and neonatal mortality in lambs. This study was designed to 1) estimate animal and flock seroprevalence of *B. ovis* in sheep across Wyoming, USA, and 2) describe epidemiologic risk factors associated with seropositive sheep and flocks. For the animal seroprevalence estimate, 2423 blood samples were collected from sheep on 18 producer-selected operations and a questionnaire about possible risk factors was distributed. For the flock seroprevalence estimate, blood samples from 82 operations were obtained, including samples from the previous 18 operations and 64 additional operations that sent samples to the Wyoming State Veterinary Laboratory for diagnostic testing. Categorical risk factors were created based on questionnaires and submission forms. Sera was analyzed using the *B. ovis* enzyme-linked immunosorbent assay.

**Results:**

Estimated true animal and flock seroprevalence were 0.53% (95% CI: 0.21–1.01%; 22/2,423) and 22.5% (95% CI: 14–32%; 18/82), respectively. Using Fisher’s exact and Mid-p exact tests to compare apparent seroprevalence with respect to possible risk factors, increased age and breed type were risk factors associated with seropositive sheep, while region and large flock size were risk factors associated with seropositive flocks.

**Conclusions:**

Results from this study suggest few sheep have been exposed to *B. ovis*, but many flocks contain at least one seropositive animal. Each region in Wyoming contained at least one seropositive animal and flock, emphasizing the importance of disease-free documentation before purchasing new sheep. Aged sheep (≥ 6 years of age) had the highest seroprevalence among age groups; hence, we propose the separation of young rams from older rams to help reduce disease spread outside the breeding season. Wool breeds (Rambouillet and Merino) may be less susceptible to *B. ovis* infection given they had the lowest animal seroprevalence of the breed types, and large flocks (> 100 breeding rams) had the highest seroprevalence of the flock size categories, likely due to more intensive management strategies that can contribute to the introduction and persistence of *B. ovis* infection in sheep and flocks.

**Electronic supplementary material:**

The online version of this article (10.1186/s12917-019-1995-5) contains supplementary material, which is available to authorized users.

## Background

*Brucella ovis (B. ovis)* is the causative agent of a sexually transmitted, infectious disease resulting in clinical or subclinical chronic disease in domestic sheep [1]. The disease was first described in Australia and New Zealand in 1953 [2, 3] and was later discovered in the United States [4]; however, disease likely occurs in most major sheep-producing countries [1]. During natural infection, disease is introduced into a flock after the reintroduction of an infected ram, and the most common route of disease spread is indirect ram-to-ram transmission via passive venereal contact with ewes during the breeding season [5–8]. Rams excrete *B. ovis* in semen intermittently for two or more years, and ewes occasionally excrete *B. ovis* in vaginal discharge and in milk near parturition; however, infection rarely persists for successive breeding seasons [7, 8]. Disease transmission also occurs directly from ram-to-ram via oral and mucosal routes outside the breeding season [7, 9].

The most common clinical sign of *B. ovis* infection is epididymitis in rams, which can be detected on a breeding soundness examination (BSE) prior to the breeding season. A proper BSE consists of a physical exam to assess body condition, scrotal circumference, presence of ulcerative posthitis, or epididymitis, and a semen evaluation by microscopy to assess spermatozoa motility, morphology, and white blood cell count [10]. However, less than 50% of rams infected with *B. ovis* develop a palpable epididymitis, and infection with other bacterial agents can cause abnormalities in spermatozoa [10]. Therefore, diagnosis with the indirect enzyme-linked immunosorbent assay (ELISA) is preferred. The *B. melitensis* Rev. 1 vaccine is a live strain vaccine that can stimulate immunity against *B. ovis* [1]; however, disadvantages of vaccine use include the development of *B. melitensis* and *B. ovis* antibodies creating interference on serological tests, and human disease caused by accidental self-inoculation [11]. For these reasons, the *B. melitensis* Rev. 1 vaccine is prohibited from use in countries affected by *B. ovis*, but free from *B. melitensis*, including the U.S. [1, 5, 12]. Antibiotic treatment is often not financially feasible; hence, a test-and-slaughter management strategy is currently the most cost-effective option for eliminating *B. ovis* in flocks in the U.S. [5].

The economic repercussions of *B. ovis* include decreased fertility in rams, lower conception rates in ewes, and a reduction in the total number of healthy lambs born. *Brucella ovis* is a concern for many U.S. sheep operations, as lamb production accounts for a high percentage of gross annual sales [13]. Furthermore, indirect reproductive and financial costs associated with *B. ovis* include the purchase of additional rams to effectively breed ewes, culling of genetically valuable rams due to infection, repetitive serological testing to control disease, and prolonged lambing seasons that can be detrimental in states with severe weather.

Research regarding the importance of *B. ovis* in domestic sheep has been done primarily on rams in major sheep-producing regions [9, 14–20], but none have estimated seroprevalence or assessed possible risk factors associated with *B. ovis* in both rams and ewes in North American flocks. The information from this study will help fill the knowledge gap regarding seroprevalence of this reproductive disease in the U.S., aid in future management decisions for producers, and give insight into the relevance and epidemiology of *B. ovis* in ewes. The objective of this study was to estimate true animal and flock seroprevalence of *B. ovis* in study populations in Wyoming, and describe epidemiologic risk factors associated with seropositive sheep and flocks.

## Results

### Animal seroprevalence and risk factors

Among all sheep tested for *B. ovis* antibodies, 22 were seropositive, resulting in an apparent seroprevalence of 0.91% (95% CI: 0.60–1.37%) and an estimated true seroprevalence of 0.53% (95% CI: 0.21–1.01%) in the study population when considering test sensitivity and specificity. Apparent animal seroprevalence and the odds of *B. ovis* exposure for potential risk factors are presented in Table 1.

**Table 1:** Apparent animal seroprevalence and odds of *B. ovis* exposure for risk factors in Wyoming, USA

Risk Factor	N	Seroprevalence^a^	Odds Ratio (95% CI)	*P* value
Region				
Northeast	298	0.39% (1/255)	Reference	–
Northwest	255	1.68% (5/298)	3.90 (0.59 to 103.51)	0.346
West	832	0.48% (4/832)	1.11 (0.15 to 30.48)	0.927
South-central	572	1.57% (9/572)	3.60 (0.66 to 90.16)	0.346
Southeast	466	0.64% (3/466)	1.51 (0.17 to 43.49)	0.927
Gender				
Rams	1477	0.95% (14/1477)	Reference	–
Ewes	946	0.85% (8/946)	0.90 (0.35 to 2.13)	0.812
Age Groups^b^				
Lambs	147	0.00% (0/147)	–	–
Yearlings	538	0.56% (3/538)	Reference	–
Adults	1337	0.60% (8/1337)	1.04 (0.29 to 5.02)	0.955
Aged	220	2.27% (5/220)	4.06 (0.95 to 21.20)	0.118
Primary Breed Type^b^				
Wool	1083	0.28% (3/1083)	Reference	–
Meat	402	1.24% (5/402)	4.44 (1.04 to 23.12)	0.044
Multi-purpose	890	1.35% (12/890)	4.73 (1.48 to 21.79)	0.014
Time of Sample Collection				
After Breeding	754	0.53% (4/754)	Reference	–
Before Breeding	1669	1.08% (18/1669)	1.98 (0.73 to 7.06)	0.191

^a^Parenthesis indicate proportion of seropositive sheep per category

^b^Indicates significant difference between seroprevalence by Fisher’s exact test at *p* <  0.05

Of the 2423 sheep sampled for this study, 12% (298/2423) were from the Northwest region, 11% (255/2423) were from the Northeast region, 34% (832/2423) were from the West region, 24% (572/2423) were from the South-central region, and 19% (466/2423) were from the Southeast region. Sheep in the Northwest region had the highest apparent seroprevalence (1.68%), but there was not a statistically significant difference between seroprevalence and the five regions (*p* = 0.113) according to the Fisher’s exact test.

Rams comprised 61% of sheep sampled in this study (1477/2423) and ewes comprised 39% (946/2423). Rams had a slightly higher apparent seroprevalence compared to ewes (0.95 and 0.85%, respectively); however, this difference was not statistically significant (*p* = 0.812) according to the Mid-p exact test.

Of the 2242 sheep sampled with known ages, 7% (147/2242) were lambs, 24% (538/2242) were yearlings, 59% (1337/2242) were adult sheep, and 10% (220/2242) were aged sheep. No lambs were seropositive in this study; therefore, we eliminated this category from further analysis. Apparent seroprevalence was 0.56% in yearling sheep, 0.60% in adult sheep, and 2.27% in aged sheep, with a statistically significant difference between seroprevalence among these age categories (*p* = 0.044) (Table 1) according to the Fisher’s exact test. Furthermore, the odds of *B. ovis* exposure in aged sheep was about 4 times that of yearling sheep.

Of the 2375 sheep sampled with known breeds, 46% (1083/2375) were wool breeds, 37% (890/2375) were multi-purpose breeds, and 17% (402/2375) were meat breeds. There was a statistically significant difference in seroprevalence between primary breed types, as 0.28% of wool breeds, 1.24% of meat breeds, and 1.35% of multi-purpose breeds were seropositive (*p* = 0.012) (Table 1) according to the Fisher’s exact test. The odds of *B. ovis* exposure in multi-purpose and meat breeds was 4.7 and 4.4 times, respectively, the odds of *B. ovis* exposure in wool breeds.

Of the 2423 samples collected in this study, 69% (1669/2423) were collected before the breeding season, and 31% (754/2423) were collected after the breeding season. Apparent animal seroprevalence of sheep sampled before the breeding season was 1.08% compared to 0.53% for sheep sampled after the breeding season, and this difference was not statistically significant (*p* = 0.191) according to the Mid-p exact test.

Two variables (age category and primary breed type) were incorporated into a multiple logistic regression model. The wool breed type and aged sheep categories had *p*-values of 0.035 and 0.036, respectively. The negative coefficient for the wool breed type and positive coefficient for the aged sheep category suggests that, if all other variables being equal, the wool breeds are less likely to be seropositive and aged sheep are more likely to be seropositive when tested for *B. ovis* antibodies.

### Flock seroprevalence and risk factors

Among all flocks tested for *B. ovis* antibodies, 18 contained at least one seropositive animal, resulting in an apparent seroprevalece of 22.0% (95% CI: 14.4–32.1%) and an estimated true seroprevalence of 22.5% (95% CI: 14.6–33.0%) in the study population when considering test sensitivity and specificity. Apparent flock seroprevalence and the odds of *B. ovis* exposure for potential risk factors are presented in Table 2.

**Table 2:** Apparent flock seroprevalence and odds of *B. ovis* exposure for risk factors in Wyoming, USA

Risk Factor	N	Seroprevalence^a^	Odds Ratio (95% CI)	*P* value
Region^b^				
Southeast	17	5.9% (1/17)	Reference	–
Northwest	25	12.0% (3/25)	1.99 (0.21 to 60.81)	0.575
Northeast	20	25.0% (5/20)	4.67 (0.62 to 134.15)	0.294
West	6	16.7% (1/6)	3.01 (0.069 to 131.62)	0.575
South-central	14	57.1% (8/14)	17.34 (2.39 to 502.01)	0.012
Flock Size^b^				
Small	24	4.2% (1/24)	Reference	–
Very Small	20	10.0% (2/20)	2.36 (0.18 to 78.42)	0.516
Medium	32	31.3% (10/32)	9.06 (1.51 to 237.89)	0.018
Large	6	83.3% (5/6)	69.00 (5.81 to > 999.99)	< 0.001
Ewe-to-ram Ratio				
Medium	9	44.4% (4/9)	Reference	–
Low	4	25.0% (1/4)	0.47 (0.01 to 6.07)	0.587
High	4	75.0% (3/4)	3.21 (0.254 to 116.89)	0.587
Purchase of Outside Sheep				
No	7	28.6% (2/7)	Reference	–
Yes	9	55.6% (5/9)	2.83 (0.35 to 32.03)	0.340

^a^Parenthesis indicate proportion of seropositive flocks per category

^b^Indicates significant difference between seroprevalence by Fisher’s exact test at *p* < 0.05

Of the 82 flocks sampled for this study, 31% (25/82) were from the Northwest region, 24% (20/82) were from the Northeast region, 7% (6/82) were from the West region, 17% (14/82) were from the South-central region, and 21% (17/82) were from the Southeast region. Flocks in the South-central region had the highest apparent seroprevalence (57%), and contrary to the animal data, there was a statistically significant difference between seroprevalence in flocks in the five regions (*p* = 0.009) (Table 2) according to the Fisher’s exact test.

Of all flocks sampled, 24% (20/82) were very small, 30% (24/82) were small, 39% (32/82) were medium, and 7% (6/82) of flocks were large. An average of two rams per very small flock (95% CI: 2–3; median: 2), seven rams per small flock (95% CI: 6–8; median: 7), 32 rams per medium flock (95% CI: 25–38; median: 28), and 216 rams per large flock (95% CI: 115–317; median: 194) were tested. Apparent flock seroprevalence was 10% in very small flocks, 4% in small flocks, 31% in medium flocks, and 83% in large flocks. There was a statistically significant difference between seroprevalence of flocks of different sizes (*p* = 0.0002) according to the Fisher’s exact test, and the odds of a seropositive flock in the medium and large flock sizes was 9.1 and 69 times the odds, respectively, of a seropositive flock in the small flock size category (Table 2).

Seventeen producers disclosed information regarding the ewe-to-ram mating ratio in their flock. Of those 17 flocks, 23.5% (4/17) possessed low ratios (1 to 29 ewes per ram), 53% (9/17) possessed medium ratios (30 to 39 ewes per ram), and 23.5% (4/17) possessed high ratios (≥ 40 ewes per ram). Flocks with low, medium, and high ewe-to-ram ratios had apparent flock seroprevalence of 25, 44, and 75%, respectively, but this difference was not statistically significant (*p* = 0.565) according to the Fisher’s exact test.

Sixteen producers disclosed information regarding the purchase or introduction of new sheep into their flock. Of those 16 flocks, 56% (9/16) were classified as open flocks, or those that possessed one or more sheep, regardless of gender or age, obtained outside the original flock between 2015 and 2016. The other 44% (7/16) of flocks were considered closed flocks, or those that did not contain any sheep obtained outside the original flock during that year. Apparent flock seroprevalence of open flocks was 56% compared to 29% for closed flocks, and this difference was not statistically significant (*p* = 0.340) according to the Mid-p exact test.

Two variables (region and flock size) were incorporated into a multiple logistic regression model, but the results did not identify any significant factors. One explanation for this result was the relatively large amount of missing data; the model eliminated 25 observations (i.e. flocks) from the analysis due to missing information.

## Discussion

### Seroprevalence of *B. ovis* in Wyoming

In Wyoming, the estimated true animal and flock seroprevalence of *B. ovis* in the study populations was 0.53 and 22.5%, respectively. Our animal seroprevalence estimate, based on serologic testing, is much lower than prevalence estimates published elsewhere, including 14.9% in Idaho and Oregon, 27.1% in Utah, 67.5% in New Mexico, and 10.0% in rams across Colorado, Wyoming, and Utah [10, 21–23]. Some of these studies used testicular palpation and bacterial culture to estimate prevalence in rams, and others relied on samples collected from rams suspected of *B. ovis* exposure or samples collected from rams chosen by producers. Thus, these factors may have led to an overestimation of true animal prevalence. Conversely, this study included samples collected from both ewes and clinically normal rams, which may explain our lower animal seroprevalence estimate in the study population.

Reduced animal seroprevalence paired with elevated flock seroprevalence suggests few animals have been exposed to *B. ovis*, but many flocks contain at least one seropositive animal. This finding coincides with other international studies. Souza et al. [24] reported animal and flock seroprevalence of 0.72 and 8.62%, respectively, while surveying 700 sheep in 58 Brazilian flocks. Sergeant [19] reported 10.8% of rams were seropositive for *B. ovis* and 32.9% of flocks contained at least one seropositive ram in New South Wales. Machado et al. [15] observed 2.89% of rams and 2.50% of flocks tested seropositive for *B. ovis* in Brazil, while Arsenault et al. [25] surveyed 250 rams from 30 Canadian flocks and found no serological evidence of *B. ovis*.

In conclusion, the low number of seropositive sheep in this study (22/2423) may suggest *B. ovis* is a negligible disease in Wyoming; however, increased seroprevalence at the flock-level (18/82) is concerning and demonstrates the need for further outreach and surveillance.

### Significant risk factors

This study reported statistically significant differences between apparent *B. ovis* seroprevalence in flocks from different regions and different flock sizes. In a similar study, Pinheiro et al. [26] uncovered differences in *B. ovis* seroprevalence when comparing 23 flocks from three regions in Brazil.

Regarding flock size, previous studies reported lower seroprevalence in small flocks compared to medium-sized flocks, and rams from large flocks were 14 times more likely to become infected than rams from small flocks [14, 20]. Our study corroborates these findings; however, one potential pitfall is the presence of confounding flock factors. Carrera Chávez et al. [14] reported significantly higher seroprevalence in rams belonging to flocks with semi-intensive production systems, which were characterized as those that graze sheep on open grassland or rangeland, offer complement feed to increase productivity, have an average flock size of 208 ± 264 sheep, and an average ewe-to-ram ratio of 41.3 ± 16.7 ewes per ram. Although we did not have a formal production system classification for flocks in this study, large flocks in the U.S. are often associated with regions and management systems that involve grazing sheep on open rangeland, resulting in interactions with other potentially infected sheep [27]. Moreover, some regions in Wyoming have more access to public land, which can be used for shared grazing, and this may influence the average flock size in these areas. Disparities in these variables (region and flock size) can influence management strategies, and in turn, influence the likelihood of *B. ovis* exposure in a flock.

No significant differences in apparent flock seroprevalence and the ewe-to-ram mating ratio were observed. Likewise, Ficapal et al. [20] did not conclude correlation between ewe-to-ram mating ratio of a flock and the bacteriological, serological, or clinical status of *B. ovis*. In contrast, Carrera Chávez et al. [14] observed highest *B. ovis* seroprevalence in rams from flocks with low ewe-to-ram mating ratios (16 to 30 ewes per ram), suggesting that *B. ovis* causes reduced fertility in rams and results in a lower capacity to service ewes. If this occurs, producers require additional rams to re-service the open ewes, therefore, decreasing the ewe-to-ram ratio (i.e., more rams needed to breed fewer ewes). One potential explanation for this conflicting result in our study is a lack of sufficient power, given we only had information from 17 flocks. While this study did not identify ewe-to-ram mating ratio as a significant risk factor, it still may be indicative of *B. ovis* infection, and breeding rams should be tested before re-servicing open ewes to prevent increased disease transmission.

No statistically significant differences were observed in apparent flock seroprevalence between operations that purchased outside sheep (i.e., open flocks) and operations that did not purchase outside sheep (i.e., closed flocks). This finding may also be an artifact of low sample size (*n* = 16). It contrasts with Santos et al. [28], who reported significantly higher *B. ovis* seroprevalence in Brazilian flocks that purchased outside animals compared to those that did not. They also reported the odds of *B. ovis* exposure in open flocks was more than six times the odds of exposure in closed flocks [28]. In our study, the odds of *B. ovis* exposure in open flocks was greater than three times the odds of exposure in closed flocks, but not significant at this level. An increased sample size in the future may corroborate Santos et al.*’s* finding.

Similar to other studies, statistically significant differences were reported between apparent seroprevalence and age groups. Some authors have concluded probability of infection merely increases with age, while others hypothesize that *B. ovis* seroprevalence correlates with sexual activity [15, 18, 20]. In this study, there were no seropositive lambs, but there was increased apparent seroprevalence in yearling and adult sheep, with the highest seroprevalence in aged sheep. This provides support for the theory that probability of infection increases with age. While the risk of *B. ovis* exposure may be highest in animals with increased sexual activity in controlled settings, highest seroprevalence in older animals in this study is likely due to the increased number of breeding seasons and numerous exposures to *B. ovis* in a natural setting. The results also suggest it may be beneficial to segregate yearling rams from adult and aged rams outside the breeding season to reduce disease spread.

Statistically significant differences between apparent seroprevalence and different breed types were observed. More specifically, the wool breeds (e.g., Merinos and Rambouillets) had lower apparent seroprevalence than other breed types. Similarly, Sergeant [19] observed Merino rams had significantly lower seroprevalence than other British breed rams. In our study, confounding factors may have played a role in seroprevalence differences. Specific breeds are often associated with certain production systems and/or flock sizes, which may influence likelihood of *B. ovis* infection [14]. A second explanation is due to variation in genetic makeup of different breeds that may influence sexual behaviors, growth rates, and disease susceptibility [20, 29].

No statistically significant differences were observed in apparent animal seroprevalence between regions, gender, or time of sample collection. Few studies have analyzed location of an animal as a risk factor associated with *B. ovis* infection, and to our knowledge, none have reported statistical significance [15, 26, 28, 30]. That said, each region of Wyoming contained at least one seropositive animal, emphasizing the importance of proper documentation of disease status before purchasing sheep, regardless of location.

Contrary to our hypothesis, there was no statistically significant difference between apparent seroprevalence in rams and ewes in Wyoming, which reinforces results from other studies [31, 32]. This finding suggests that, when sampled on a larger scale, similar proportions of rams and ewes test seropositive; it also supports the fact that, once introduction occurs, *B. ovis* spreads to both rams and ewes. Thus, even if breeding rams are tested and seropositive rams culled on an annual basis, *B. ovis* may still be maintained by ewes in the flock.

Lastly, we hypothesized apparent seroprevalence would be significantly higher after the breeding season compared to before the breeding season because *B. ovis* is sexually transmitted during the breeding season [7, 8, 33]. In contrast, we observed higher apparent seroprevalence before the breeding season than after the breeding season. Explanations for this finding may include: 1) infection and transmission of *B. ovis* is occurring more often directly from ram-to-ram before the breeding season, 2) there is a lack of seroconversion in animals after *B. ovis* exposure, 3) samples were collected outside the optimal ELISA detection window (i.e., 19–36 days after *B. ovis* exposure) [34], or 4) high correlation of the variables, gender and time of sample collection, relative to the breeding season. The majority of samples collected before the breeding season were from rams and most samples collected after the breeding season were from ewes; therefore, we could not draw strong conclusions about seropositivity and time of sample collection relative to the breeding season alone. To our knowledge, no previous studies have considered time of sample collection relative to breeding season as a possible risk factor for seropositivity, but a more comprehensive look into this variable may be useful in the future.

### Potential bias

The initial sampling frame for apparent animal seroprevalence was a list of Wyoming sheep producers obtained from the Wyoming Woolgrower’s Association. While this sampling frame may not be truly representative of all sheep in Wyoming, it was our best available resource. We offered free serologic testing to producers in exchange for cooperation in the study, generating bias towards producers suspecting disease or otherwise interested in disease surveillance. Sheep included in the apparent animal seroprevalence estimate were a convenient subset from 18 producer-selected flocks and represented only a small portion of all breeding sheep in Wyoming. Furthermore, information about sheep and flocks were subject to producers’ discretion. Additional flocks included in the apparent flock seroprevalence estimate were ones belonging to producers that sent samples to WSVL for diagnostic testing due to suspected disease or routine annual testing. The latter (i.e., routine annual testing) creates bias towards the inclusion of likely seronegative flocks because of the producers’ adherence to certain disease management guidelines, while the former (i.e., suspected disease) creates bias towards the inclusion of likely seropositive flocks. We assumed the total number of ram samples sent in for diagnostic testing at WSVL were all breeding rams on the operation, and we used that number to classify flocks into size categories. This could have created miscalculations during our flock size analysis. Diagnostic sensitivity and specificity of the ELISA used was not 100%, resulting in the possibility of false positive and negative results in this study. In a flock of 500 sheep with known *B. ovis* infection, the ELISA would correctly identify 482 infected sheep and misdiagnose 18 sheep as false negatives. However, in a flock of 500 *B. ovis*-free sheep, this ELISA would misdiagnose two sheep as false negatives, which is problematic for disease control. For apparent animal and flock seroprevalence, we considered indeterminate results as seronegative, as to not over-estimate the number of seropositive results. Finally, the low number of seropositive results (22 sheep and 18 flocks) combined with missing data limited our interpretation of the multiple logistic regression models.

## Conclusions

This study was the first to estimate true flock seroprevalence of ovine brucellosis in a study population in Wyoming, with 22.5% of flocks containing at least one seropositive animal. Estimated true animal seroprevalence of *B. ovis* in the study population (0.53%) was lower than in other studies conducted in the western U.S. The most important risk factors associated with seroprevalence included age and breed type for animal seroprevalence, and region and flock size for flock seroprevalence. Animal seroprevalence gradually increased from yearling to aged sheep, suggesting older sheep that have undergone numerous breeding seasons are more likely to test seropositive and may continue to infect others due to the chronic nature of this bacterial infection. The separation of young rams from older rams outside the breeding season to limit the spread of *B. ovis* is a possible preventive strategy. Wool breeds had lower animal seroprevalence compared to other breed types, suggesting Rambouillet and Merino breeds are either less susceptible to *B. ovis* infection or additional confounding variables contribute to this difference. Flock seroprevalence was different among regions in Wyoming, demonstrating that flocks in certain areas likely share other characteristics that influence *B. ovis* seroprevalence. Lastly, flock seroprevalence generally increased as flock size increased, which may be attributed to variations in management strategies that depend on overall flock purpose. Other variables did not have a clear influence on seroprevalence of *B. ovis* in this study; however, maintenance of open flocks and lower ewe-to-ram ratios may be indicative of *B. ovis* infection in a flock.

## Methods

The University of Wyoming Institutional Animal Care and Use Committee (IACUC) approved the study and sampling method (protocol #200150622KS00178–01).

### Study population and sample collection

**Fig. 1:**
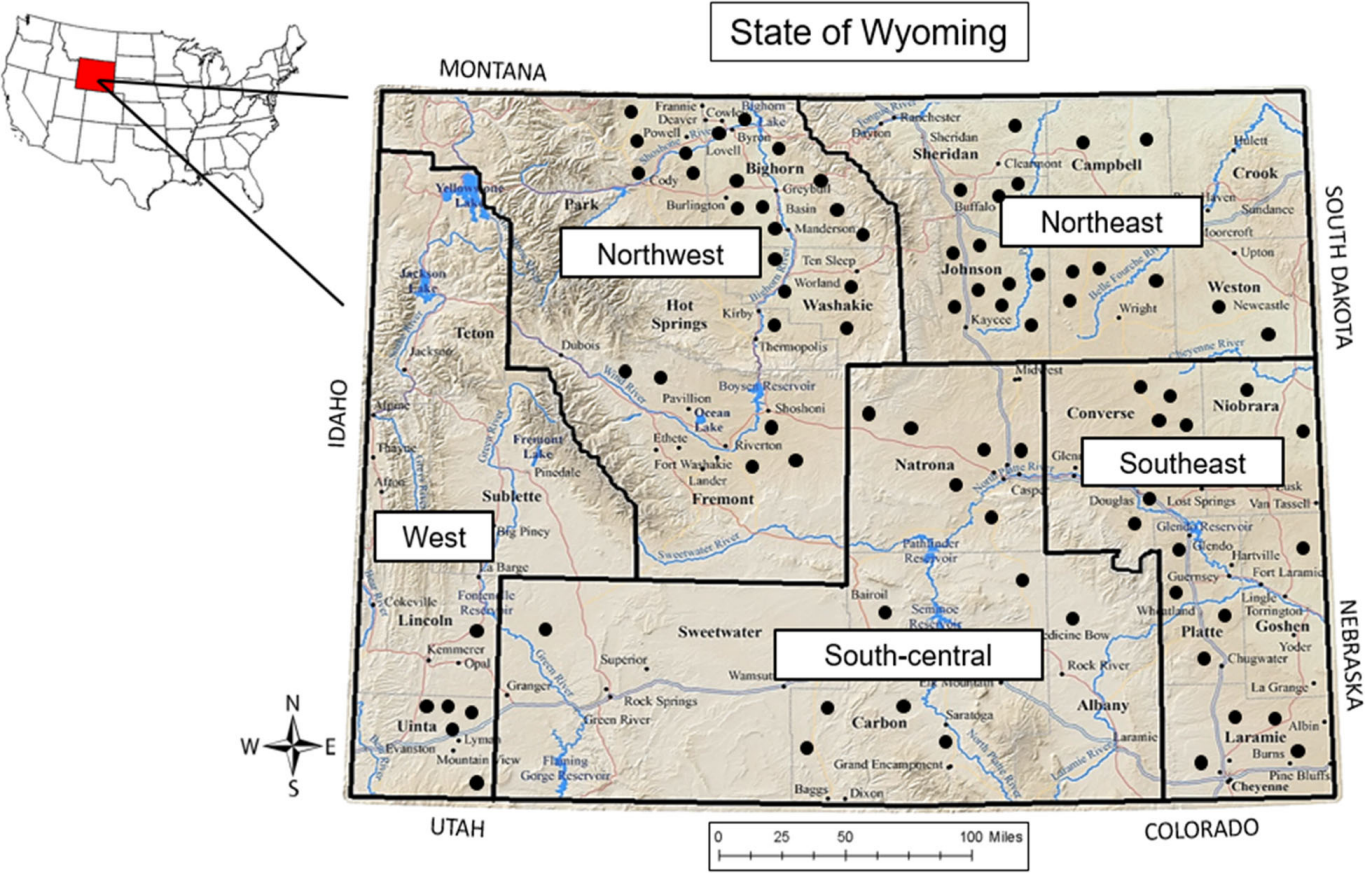
Study area and approximate locations of sampled operations for estimated *B. ovis* flock seroprevalence

The study area was the state of Wyoming, USA, which is approximately 253,819 km^2^ in size with suitable land for sheep production ranging from 900 to more than 3,000 m in elevation. Figure 1 shows the study area and approximate locations of sampled operations for estimated *B. ovis* flock seroprevalence) [13]. The state had 293 sheep operations in 2015 and ranked third in the nation for the largest breeding sheep population with 210,000 ewes and 7,000 rams [35, 36]. Five geographic regions were created based on agricultural districts used by the United States Department of Agriculture (USDA) and the National Agricultural Statistics Services (NASS).

Though the total number of sheep operations in Wyoming was known, contact information was not available. Hence, the starting sampling frame was a list of 82 Wyoming sheep producers obtained from the Wyoming Woolgrower’s Association [37]. Producers were contacted by telephone, email, or brochures sent in the mail and were offered free serological testing for a portion of their flock in exchange for cooperation in the study. Eighteen producers expressed interest, and a questionnaire approved by the University of Wyoming’s Institutional Review Board (IRB) was utilized to obtain additional information about flock size, predominant breeds, and breeding practices. Refer to “2015-2016 Sheep Brucellosis Survey” in the Additional file 1 for a full list of questions.

Permission to enter property and collect blood samples was obtained from the 18 operations prior to sample collection, and sampling dates were chosen based on accessibility of sheep between August 2015 and May 2016. Sheep included in the animal seroprevalence estimate were a convenient subset of available ewes and, if possible, all breeding and yearling rams from 18 producer-selected operations in Wyoming. Yearling rams and ram lambs that had not undergone breeding, but were housed with mature, breeding rams were included in the initial study population. If rams were not available for sampling at the time of blood collection (e.g., sampling occurred during pregnancy checking of ewes), results from ram samples submitted to the Diagnostic Serology Lab at the Wyoming State Veterinary Laboratory (WSVL) by the same producer between January 2015 and May 2017 were used. If ewes were not available for sampling at time of blood collection (e.g., sampling occurred during BSEs of rams), results from ewe samples submitted to WSVL by the same producer were used.

During sample collection, a properly trained student, producer, veterinarian, or veterinary technician drew 5–10 mL of blood via jugular venipuncture of physically healthy sheep using 10 mL blood collection tubes (BD Vacutainer, Franklin Lakes, NJ, USA) and needle holders (BD Vacutainer, Franklin Lakes, NJ, USA) with disposable 1 ½ inch, 18–20 gauge needles (Greiner Bio-One, Kremsmünster, Austria). Relevant information was recorded for each sheep (i.e., location, gender, age, breed, and date of sample collection) at the time of blood collection. Blood was allowed to clot before transportation back to WSVL on ice. Sera was separated from blood samples using a centrifuge, decanted into labeled tubes, and stored at − 20 °C until serologic testing.

The sample size required for each operation was calculated post-collection after questionnaires revealed the total number of breeding rams and ewes in the flock [38]. For operations in which breeding ewes and rams were available for sampling (*n* = 9), we used the total number of breeding sheep for population size, expected true seroprevalence of 10% [10], acceptable error of 5%, confidence level of 95%, and test sensitivity and specificity of 96.3 and 99.6% [39], respectively. For operations in which only rams were available for sampling (*n* = 9), we used the total number of breeding rams in the flock for population size and the same inputs above. In some operations, the number of samples collected was not equivalent to the required sample size because samples were collected prior to the sample size calculations. A total of 2423 sheep were used to estimate true seroprevalence in the study population, as shown in Table 3.

**Table 3:** Total blood samples collected from 2423 sheep in 18 flocks in Wyoming, USA

Flock	Total number of sheep		Number of blood samples		Required sample size	Total samples
	Rams	Ewes	Rams	Ewes		
1	50	1600	23	N/A	38	23
2	75	2300	50	196	142	246
3	10	35	10	N/A	10	10
4	210	6000	185	157	148	342
5	15	120	9	16	72	25
6	40	1000	40	N/A	32	40
7	105	2500	90	144	143	234
8	12	400	9	N/A	12	9
9	400	8000	393	64	149	457
10	100	2000	66	N/A	61	66
11	202	6000	202	N/A	87	202
12	55	2100	55	N/A	41	55
13	25	80	15	20	62	35
14	70	730	67	69	128	136
15	35	950	32	92	135	124
16	250	9500	187	188	149	375
17	50	1250	35	N/A	38	35
18	NA	NA	9	N/A	NA	9
Total			1477	946	1447	2423

N/A indicates information and/or samples were not available

### Additional flock inclusion criteria

To bolster confidence in the flock seroprevalence estimate in the study population, we used the samples from the 18 producer-selected operations from above, in conjunction with samples that other Wyoming producers submitted to the Diagnostic Serology Lab at the WSVL for *B. ovis* testing between January 2015 and May 2017. These additional flocks were not used in the animal seroprevalence estimation and risk factor analysis because the questionnaire was not distributed to these producers. Additional flocks eligible for inclusion in the flock seroprevalence estimation required a WSVL submission form indicating producer surname, producer and/or veterinary clinic address within Wyoming, and a minimum of two samples from different sheep in the same flock. The flock was excluded if there was no producer surname or if the surname matched one of the 18 producer-selected operations already included. Some forms lacked a producer address; however, if the veterinary clinic listed on the form was located within the state, an assumption was made that the flock resided in the same region. A minimum requirement of two samples on each submission form allowed the inclusion of small flocks whose main purpose is club lamb or 4-H lamb production. Some producers submitted multiple forms for diagnostic testing for their flocks, and to reduce confounding, only one form per producer surname was considered. If a single producer submitted multiple forms, the submission form with the most samples was used to achieve a more accurate estimation of overall flock size. If a single producer submitted multiple forms containing the same number of samples, the form that was submitted first was used.

The sample size required for the flock seroprevalence estimate was 173 when using a total population size of 293, expected true seroprevalence of 50%, acceptable error of 5%, confidence level of 95%, and test sensitivity and specificity of 96.3 and 99.6% [39], respectively. That said, we had access to sera from 82 Wyoming flocks (18 producer-selected flocks and 64 flocks from WSVL submission forms), which we used to estimate true flock seroprevalence in the study population.

### Classification and description of risk factors

Continuous and categorical information obtained at the time of sample collection, via questionnaire, or WSVL submission forms was utilized to create categorical risk factors for animals and flocks. Possible risk factors evaluated for animals included region, gender, age group, primary breed type, and time of sample collection relative to the breeding season. Possible risk factors evaluated for flocks included region, flock size, ewe-to-ram mating ratio, and the introduction of new sheep into the flock.

For regional classification, both animals and flocks were classified into one of five categories based on the county where breeding occurred for the 18 producer-selected operations, and the location of producer and/or veterinary clinic address for the 64 operations that sent samples to WSVL for *B. ovis* testing. Sheep were classified into one of two gender categories. Sheep were classified into one of four categories based on known age (i.e., ear tag or producer knowledge) or estimated age (i.e., number or condition of teeth). Age categories include lambs (< 1 year of age), yearlings (≥1 and < 2 years of age), adults (≥2 and < 6 years of age), and aged sheep (≥ 6 years of age), which was similar to categorization in Van Metre et al. [10] and Wiemer and Ruttle [23]. Sheep were classified into one of three categories based on the primary purpose of the breed or crossbreed. Sheep breeds can be dual purpose, but most excel in the production of wool, meat, or dairy. There were no dairy breeds in this study; therefore, categories included wool breeds, meat breeds, and multi-purpose breeds. Wool breeds include Rambouillet and Merino purebreds or crossbreeds. Meat breeds include Dorset, Hampshire, Suffolk, and Texel breeds or crossbreeds. Multi-purpose breeds include Columbia, Finn sheep, and Targhee breeds or crossbreeds. Sheep were classified into one of two categories based on date of sample collection relative to known breeding months, and include sheep sampled before the breeding season and sheep sampled after the breeding season.

Flocks were classified into one of four flock size categories based on the known number of rams in the flock for the 18 producer-selected operations, and the number of ram samples tested at WSVL for the 64 additional operations. Breeding soundness exams and serological testing for *B. ovis* infection typically occur once per year prior to the breeding season. Therefore, an assumption made was that the total number of ram samples submitted to WSVL for testing was equivalent to the total number of breeding rams in the flock at the given time. Flock size categories included very small (2–3 breeding rams), small (4–10 breeding rams), medium (11–100 breeding rams), and large (> 100 breeding rams). Flocks were classified into one of three categories based on the number of ewes an average ram can breed in one season. This number depends on multiple aspects, including the age and breeding experience of the ram, the environment in which the ram is working, and the number of ewes in the flock. We based our categories on recommendations from producers and categories from similar studies. Flock categories for ewe-to-ram mating ratios included low (1 to 29 ewes per ram), medium (30 to 39 ewes per ram), and high (≥40 ewes per ram). Flocks were classified into one of two categories based on whether the producer purchased and introduced new sheep into the flock that year (i.e., open flock) or not (i.e., closed flock).

### Serologic testing and quality control

Serologic testing was conducted at WSVL using the National Veterinary Services Laboratory (NVSL) *B. ovis* ELISA. Currently, this is the only ELISA licensed in the U.S. for detection of *B. ovis* antibodies in sheep and goat serum, and diagnostic sensitivity and specificity is reported to be 96.3 and 99.6%, respectively [39]. The antigen used for the assay is a hot-saline water soluble extract of the REO 198 strain prepared according to the World Organization for Animal Health recommendations by the Animal and Plant Health Inspection Service and the NVSL (APHIS-NVSL, Ames, IA, USA); controls include “high positive” sera, “low positive” sera, and “negative” sera [1]. NVSL personnel tested the antigen and control sera for potency and sterility, and all antigen and controls used in this study were from the same lot number.

At WSVL, the same two persons prepared and performed all assays. Sample sera were run in duplicate and control sera in triplicate with additional “low positive” and “negative” controls on the back of the plate to ensure consistent development during the assay. During plate preparation, polystyrene micro-titer plates (Nunc A/S, Roskilde, Denmark) were coated with an antigen dilution in carbonate buffer, incubated, and blocked with bovine serum albumin solution (KPL, Inc., Gaithersburg, MD, USA). Prior to running the assay, conjugated Protein G-Biotin from *Streptococcus spp.* (Sigma-Aldrich Corporation, St. Louis, MO, USA), Vectastain conjugate (Vector Laboratories, Burlingame, CA, USA), Blue Phosphatase substrate (KPL, Inc., Gaithersburg, MD, USA), and APstop solution (KPL, Inc., Gaithersburg, MD, USA) were prepared according to the manufacturers’ instructions. During assay procedure, sera, conjugated Protein G-Biotin, and Vectastain conjugate were incubated at 37 °C in a humidified chamber inside a digital display incubator (Boekel Industries, Inc., Feasterville, Trevose, PA, USA) for 45 min, 35 min, and 25 min, respectively. After each incubation, the plate was washed with a high salt wash solution, excess liquid emptied in the sink, and the plate tapped onto absorbent material to remove any non-specifically bound proteins or antibodies. The Blue Phosphatase substrate incubation step was performed at room temperature on a microplate shaker (Lab-line Instruments, Inc., Melrose Park, IL, USA), and color development stopped when the low positive control average reached an optical density (OD) between 0.350–0.500 at a wavelength of 620 nm on a plate reader (TECAN, Männedorf, Züich, Switzerland; BioTek Instruments, Winooski, VT, USA). The average OD for each sample and control was calculated, and the average sample to positive (S/P) ratio used to determine sample status. An S/P ratio of greater than 0.75 was positive, greater than or equal to 0.40 and less than or equal to 0.75 was indeterminate, and less than 0.40 was negative.

If the average OD for control sera on the front and back of the plate was outside the expected acceptable range of values for that particular lot, the plate was considered invalid and each sample re-tested. If control sera yielded ODs with a difference of greater than 20%, the well with the largest variance was excluded, the percent difference recalculated, and if acceptable (i.e., less than 20%), the ODs were then used to calculate an average for the control. If sample sera yielded two ODs with a difference of greater than 20%, the sample was re-tested. When sample sera yielded an indeterminate or positive result, the sample was re-tested to ensure minimal reporting of false positive or false indeterminate results. If a re-tested indeterminate or positive sample had the same result, the average of the S/P ratios was calculated. If a re-tested indeterminate or positive sample had a different result, the sample was re-tested a third time, and the average of the two S/P ratios with the least amount of variance was calculated.

### Statistical analysis

Sheep with seropositive test results were considered positive for *B. ovis* antibodies, and sheep with indeterminate and negative test results were negative for *B. ovis* antibodies. Flocks were considered positive if one or more sheep within the flock had a seropositive result. The apparent animal and flock seroprevalence was calculated, and the Rogan and Gladen (1978) formula was used to estimate true seroprevalence in the animal and flock populations using a sensitivity of 96.3% and specificity of 99.6% [39, 40]:

$$ True\ prevalence=\frac{\left[ Apparent\ prevalence+\left( Specificity-1\right)\right]}{\left[ Sensitivity+\left( Specificity-1\right)\right]} $$

For apparent seroprevalence, we calculated 95% confidence intervals (CI) using Wilson-Score intervals. For estimated true seroprevalence in the study populations, we calculated 95% CIs using Blaker’s intervals, as recommended by the authors of EpiTools epidemiological calculators provided by AusVet online [38, 41].

For variables that had two levels within the category (i.e. gender, time of sample collection, and purchase of outside sheep), we used the Mid-p exact test with odds ratios and 95% CIs to compare overall apparent seroprevalence. For variables that had more than two levels within the category (i.e. region, age category, breed type, flock size, and ewe-to-ram ratio), we used the Fisher’s exact test to compare overall apparent seroprevalence followed by the Mid-p exact test with odds ratios and 95% CIs to further compare seroprevalence between levels within each variable. Individual sheep or flocks were excluded from calculations if they lacked the necessary information (e.g., age, breed, ewe-to-ram ratio, etc.) needed for the risk factor analyses. For example, if one sheep had information regarding its gender and breed, but lacked information regarding its age, we included that sheep in the gender and breed analysis and excluded it from the age analysis. Values of *p* <  0.05 were considered statistically significant, and only significant variables were incorporated into a multiple logistic regression model. For variables with multiple levels, *p*-values have been adjusted using the false discovery rate (FDR) method. The statistical analyses were performed using RStudio 1.0.136 [42] within R version 3.4.3.

## Additional file

Additional file 1: 2015-2016 Sheep Brucellosis Survey. (DOCX 589 kb)

## Data Availability

The datasets generated and/or analyzed during the current study are not publicly available due to cooperating producer privacy and confidentiality, but are available from the corresponding author on reasonable request.

## Abbreviations

*B. sheep*: *Brucella ovis*
BSE: Breeding soundness exam
CI: Confidence interval
ELISA: Enzyme-linked immunosorbent assay
IACUC: Institutional Animal Care and Use Committee
IRB: Institutional Review Board
WET: National Agricultural Statistics Service
NVSL: National Veterinary Services Laboratory
FROM: Optical density
S/P ratio: Sample to positive ratio
USDA: United States Department of Agriculture
WSVL: Wyoming State Veterinary Laboratory
