# Supplementary material for: Seroprevalence and risk factors of Brucella ovis in domestic sheep in Wyoming, USA
Source: BMC Vet Res. 2019 Jul 15;15:246. doi: 10.1186/s12917-019-1995-5 (PMC6631759; doi:10.1186/s12917-019-1995-5)
Supplement: Supplementary file 1 — 2015-2016 Sheep Brucellosis Survey. (DOCX 589 kb) [file 12917_2019_1995_MOESM1_ESM.docx]

| 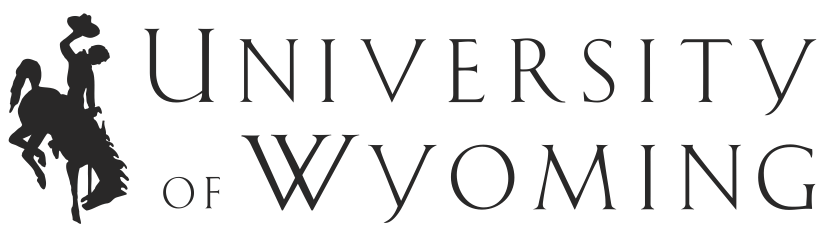 | **Molly Elderbrook M.S. Graduate Student Dept. of Veterinary Sciences**  **1174 Snowy Range Road Laramie, WY 82070**  **(262) 210-4557**  [**melderbr@uwyo.edu**](mailto:melderbr@uwyo.edu) |
| --- | --- |
| **Producer/Operation Name:**  2015-2016 Sheep Brucellosis Survey | |
| **Best Phone #:** | |
| **Email (optional):** | |

1. **What is the predominant breed(s) or cross-breed(s) of sheep on your operation?**
   1. **Breed(s)/Breed crosses :**
2. **How many replacement sheep of mature/breeding age were purchased in 2015?**
   1. **None (we keep a closed flock)**
   2. **Replacement ewes:**
   3. **Replacement rams:**
3. **Do you test replacement rams for *B. ovis* or ensure *B. ovis*-free status prior to purchase?**
   1. **Yes**
   2. **No**
4. **Approximately how many mature/breeding sheep were present on your operation in 2015?**
   1. **Mature/breeding ewes:**
   2. **Mature/breeding rams:**
5. **What was your average Ram: Ewe ratio during the 2015 breeding season?**
   1. **Ram: Ewe Ratio:**
6. **What month(s) and location did breeding occur during the 2015 season?**
   1. **Breeding month(s):**
   2. **County where breeding occurred:**
7. **What month(s) did lambing take place during the 2015-2016 season?**
   1. **Lambing month(s):**
8.
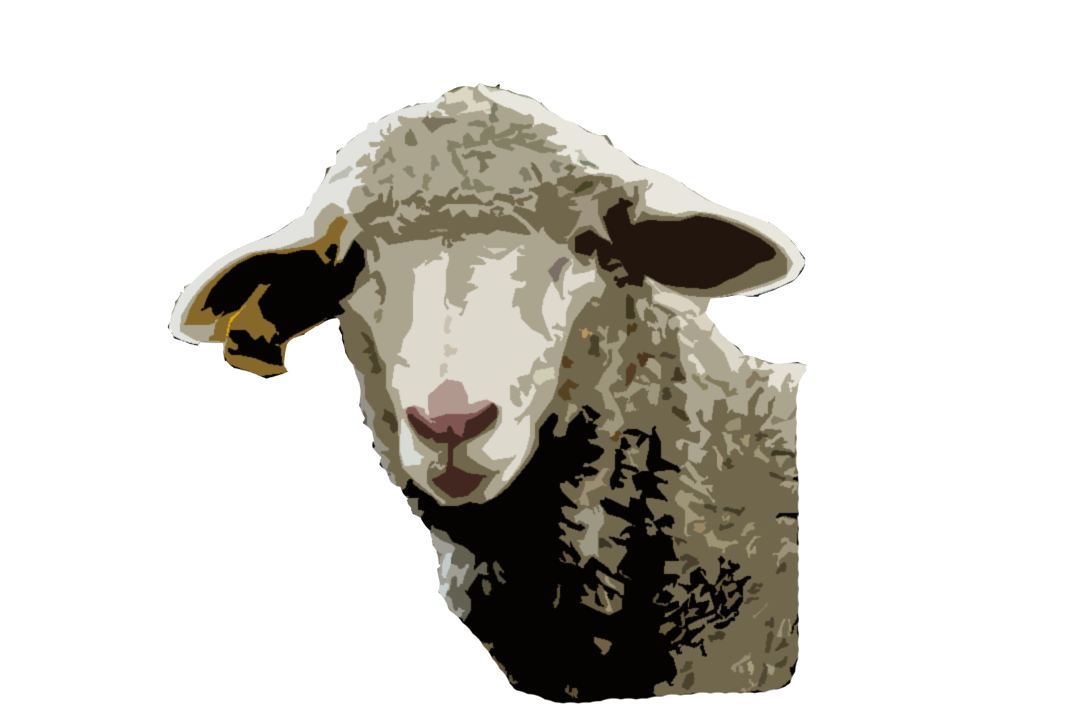
**Can the University of Wyoming use the samples collected in this study for future research?** □ **YES** □ **NO**

    The University of Wyoming and I would like to sincerely
    thank you for your cooperation with this study. To show
    our gratitude, we would like to send you a free gift.

    ***Please provide your mailing address in the box provided:***

**MAILING ADDRESS:**
